# A modified rabbit model of tracheal stenosis and a household endoscope. More simplicity and accessibility^1^

**DOI:** 10.1590/ACB351104

**Published:** 2020-12-18

**Authors:** Guoying Zhang, Jianming Wang, Yiming Zeng

**Affiliations:** I PhD, Department of Pulmonary and Critical Care Medicine, Respiratory Medicine Center of Fujian Province, The Second Affiliated Hospital of Fujian Medical University, and Department of Emergency Medicine, Quanzhou First Hospital of Fujian Medical University, Quanzhou, Fujian, China. Substantive scientific and intellectual contributions to the study; conception and design; acquisition, analysis and interpretation of data; histopathological examinations; statistics analysis; manuscript preparation and writing; final approval; II Master, Department of Pulmonary and Critical Care Medicine, Respiratory Medicine Center of Fujian Province, The Second Affiliated Hospital of Fujian Medical University, and Department of Emergency Medicine, Quanzhou First Hospital of Fujian Medical University, Quanzhou, Fujian, China. Substantive scientific and intellectual contributions to the study, manuscript preparation and writing, critical revision, final approval.; III Master, Full Professor, Department of Pulmonary and Critical Care Medicine, Respiratory Medicine Center of Fujian Province, The Second Affiliated Hospital of Fujian Medical University, Quanzhou, Fujian, China. Substantive scientific and intellectual contributions to the study, conception and design, manuscript preparation and writing, critical revision, final approval.

**Keywords:** Tracheal Stenosis, Disease Models, Animal, Pathologic Processes, Endoscopes, Rabbits

## Abstract

**Purpose:**

To develop a simpler animal model for benign tracheal stenosis and introduce a low-cost household endoscope for postmodeling endotracheal evaluation.

**Methods:**

Twenty rabbits were randomly divided into a model group (15 rabbits, subjected to transoral nylon brush scraping of the trachea) and a mock group (5 rabbits, merely exempted from scraping), a household endoscope was then introduced for weekly endoscopic examination. Meanwhile, other 15 rabbits (modeling like the model group) underwent batch tracheal resection at different postintervention times for pathological analysis.

**Results:**

The model group presented a low mortality and few complications. The endoscope could obtain adequate images for stenosis assessment, which showed that the models presented homogeneous injury after scraping and developed a mature scar stricture at 28 days postoperatively with a mean stenosis degree of 65.9%, and 71.4% (10/14) above Myer–Cotton’s grade II. The pathological findings were consistent with the clinicopathological process of human. No stenosis was found in mock group.

**Conclusion:**

The modified model is simpler, minimally invasive and reliable, while the household endoscope is competent for model’s follow-up, providing easily accessible and useful tools for facilitating more extensive studies of benign tracheal stenosis.

## Introduction

Recently, with the widespread application of respiratory support techniques in the management of critical patients, the prevalence of tracheostomy and the development of interventional pulmonology, the morbidity of benign central airway stenosis has greatly increased^1^. The treatment modalities for benign airway stenosis mainly include tracheal stenosis resection and reconstruction, balloon dilatation, stent implantation, thermal ablation, cryotherapy, topical remedy therapy, and intracavitary brachytherapy^2^. However, due to the high restenosis rate, the long-term effect is limited^3^.

The establishment of stable and reliable animal models of benign tracheal stenosis is of great significance for the in-depth study of its pathogenesis and development of new preventive strategies and therapies. Various methods have been developed to create animal models of tracheal stenosis^4^–^8^, and each model has its advantages and disadvantages in terms of technical factors, equipment requirement, cost, animal accessibility, animal mortality, and reliability, with no ideal model suitable for wide popularization.

In view of this, this study referred to and modified the reported modeling methods, aimed to develop a reliable model that not only well interprets the clinicopathological process in humans, but also is simpler, without requirement for elaborate techniques and expensive equipment, and is minimally invasive, with survival advantage. Moreover, this study attempted to utilize a low-cost household endoscope for the observation and evaluation after modeling with a view to providing a more accessible and useful tool for the study of the model, especially for researchers in numerous developing countries who lack facilities and funding, thus facilitating broader research into benign tracheal stenosis.

## Methods

The study protocol was approved by the Committee on Animal Research of The Second Affiliated Hospital of Fujian Medical University, Quanzhou, China (Ethical Committee 2017 No. 9).

Thirty-five male New Zealand white rabbits (SLAC Laboratory Animal Co., Ltd, Shanghai, China), weighing 4.0 – 5.0 kg (8 months of age), were used in the experiment and had free access to food and water. The animals were adaptively fed for 1 week before the experiment. The main surgical procedures of the experiment were performed by the same researcher. All animal procedures were conducted in accordance with the guidelines published in the *Guide for the care and use of laboratory animals* (DHEW publication NIH85-23, 2010 Revision, Office of Science and Health Reporting, DRR/NIH, Bethesda, MD, USA).

### Establishing rabbit model of tracheal stenosis

**Figure 1:**
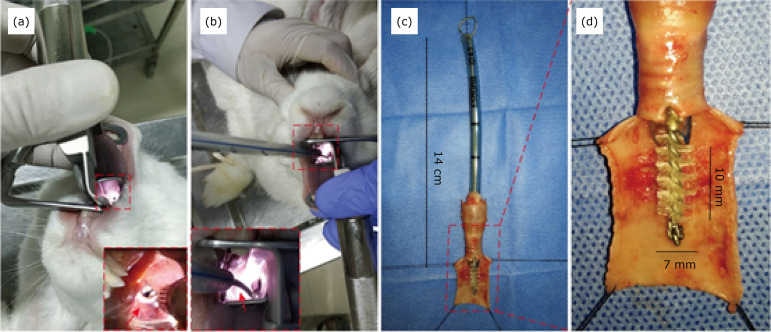
Procedure for establishing a tracheal stenosis rabbit model. (a) Exposure of glottis after raising the epiglottis (*red arrow*) with the neonatal laryngoscope. (b) A guide wire (*red arrow*) guided the orotracheal intubation, (c, d) A nylon brush was passed through the tracheal catheter to scrape the rabbit trachea (simulation intervention on an *ex vivo* rabbit trachea dissected longitudinally).

Twenty New Zealand rabbits were randomly divided into a model group (n = 15) and a mock group (n = 5). They were anesthetized by injection of pentobarbital sodium (30 mg/kg, Sinopharm Chemical Reagent Co., Ltd., Shanghai, China) into the marginal auricular vein, retaining their spontaneous breathing, and were then fixed on the operating table in a prone position. After the oropharynx of the rabbit was opened with a mouth gag (500069 BIORTHO, UK), 2% lidocaine was sprayed on the throat to prevent laryngospasm, and the neonatal direct laryngoscope (MJ-II/D-102, Maijun Medical Technology, Jiangsu, China) was inserted to press the root of the rabbit tongue, to raise the epiglottis and make the glottis visible (Fig. 1a). Under direct vision, an 8-Fr guide wire for orotracheal intubation (Tuoren Medical Technology, Henan, China) was inserted into the trachea through the glottis, and a truncated 14-cm-long tracheal catheter (ID 4.5#, Yixin Medical Technology, Henan, China) was intubated under the guidance of the guidewire, with its end aligned to the rabbit incisor (Fig. 1b). In the mock group, the tracheal catheter was then removed without further intervention, so that the effect of intubation on rabbit trachea could be evaluated separately. In contrast, in the model group, a hard nylon brush (bristle diameter, 0.2 mm; brush out-diameter, 7 mm; hair length, 10 mm: self-made) was then passed through the tracheal catheter and pulled out after rotary scraping the tracheal wall 10 times (Fig. 1 c, d). Then the rabbit position was adjusted to Trendelenburg position immediately, and posture drainage combined with negative pressure suction was adopted to prevent hemorrhage from causing airway asphyxia. Finally, the tracheal catheter was removed when there were no obvious signs of airway hemorrhage and the rabbit breath smoothly. All animals were repositioned for observation after anesthesia recovery. Complications such as cyanosis, dyspnea, and hemoptysis should be observed during the intervention. Moreover, besides the survival of the animals as the main postoperative concern, abnormal symptoms such as oral and nasal secretions, cough, hemoptysis, cyanosis, dyspnea, and mental, dietary and activity states of the animals were also observed and recorded.

### Utilizing a household endoscope for follow-up observation and measurement of stenosis

**Figure 2:**
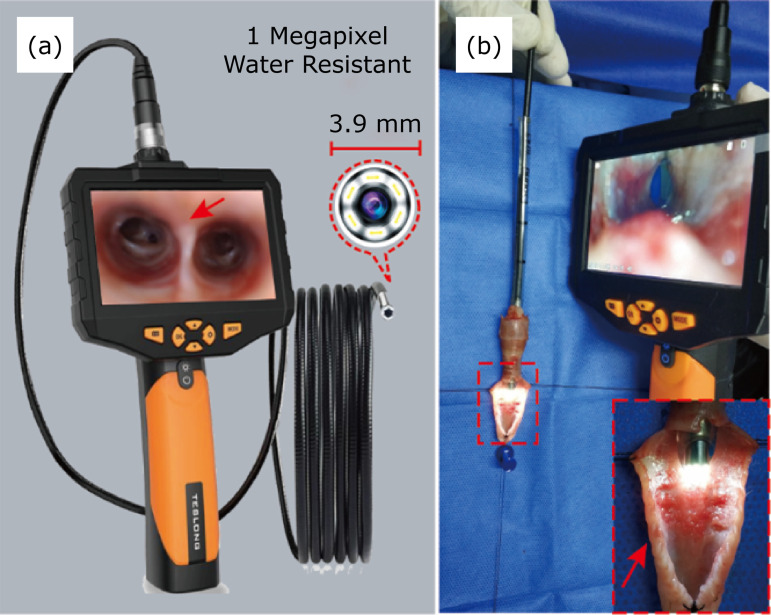
Utilization of a handy endoscope. (a) Property of the endoscopy and representative endoscopic photography (*red arrow* indicates tracheal carina), (b) Observation of the rabbit tracheal lumen with the endoscope (simulation intervention on an *ex vivo* rabbit trachea dissected longitudinally [*red arrow*]).

At different times (immediately and days 7, 14, 21, and 28) after intervention, the rabbits were anesthetized and intubated as mentioned above, followed by an alcohol-sterilized 3.9-mm-outer-diameter household endoscope (NTS300-OD3.9mm Teslong, Shengzhen, China) inserted into their tracheas through the tracheal catheter (Fig. 2). Then, endoscopic photography was performed at the beginning of the stenosis or 1 cm above the nearest normal tracheal ring to calculate the tracheal lumen area. The photographs were digitally analyzed according to pixel size using the Adobe Photoshop software (version CC; Adobe Systems, San Jose, California). The degree of stenosis was calculated using the following formula: degree of stenosis = (1 - minimum lumen area/nearest normal area) × 100%. The degree of stenosis was indexed according to Myer–Cotton’s grading system, as follows: grade I, lumen obstruction < 50%; grade II, lumen obstruction of 51–70%; grade III, lumen obstruction > 70% but still visible lumen; grade II, complete airway occlusion. In general, a degree above grade II indicates successful modeling.

### Gross examination and histological assessment

To evaluate the pathological changes over time after modeling, another 15 New Zealand rabbits were subjected to the same modeling process like the model group. They were then euthanized in batches at different postoperative times (immediately and days 7, 14, 21, and 28), and their tracheas were excised for gross examination. Subsequently, the excised tracheas were processed with standard hematoxylin-eosin staining and Masson’s staining, respectively, and the structural changes of the tracheal wall, inflammatory cell infiltration, vascular proliferation and fibrosis were observed under an optical microscope (Eclipse Ti2, Nikon).

### Statistical analysis

GraphPad Prism 8 software was used in the statistical analysis and plotting. All measures were expressed as mean ± standard deviation (*x* ± s), and data were first analyzed for normality and chi-square distribution, and the independent sample t-test was used for comparisons between the two groups. A p-value < 0.05 indicated statistically significant difference.

## Results

### Complications and mortality

The technical success rate of orotracheal intubation and nylon brush scraping was 100% with no immediate death or major complications related to the procedure, and the procedure lasted for < 15 min. The animal recovered quickly after intervention and immediately fed and drank as usual. It was not until 7 days postoperatively that some rabbits showed decreased appetite and progressive shortness of breath or stridor. Finally, three rabbits in the model group died day 6, 17, and 27 days postoperatively, and pathological examination was conducted immediately after death.

In the first dead rabbit, a trachea blocked by massive purulent white sputum with pulmonary consolidation showing “lobular pneumonia” in hematoxylin-eosin (H&E) staining, instead of fatal tracheal hyperplasia, was found. Thus, the cause of death was considered as “pulmonary infection”. Gross examination of the other two deaths, which exhibited progressive wheezing before their deaths, presented severe stenosis (85 and 90%, respectively) without any fatal lesions. Thus, the cause of their deaths was presumed to be airway obstructive asphyxia caused by severe airway stenosis. To say the least, the nonstenosis mortality rate of the model group was 1/15 (6.7%). In contrast, there was no death or abnormal symptoms in the mock group throughout the whole study period.

### Endoscopic observation and measurement

**Figure 3:**
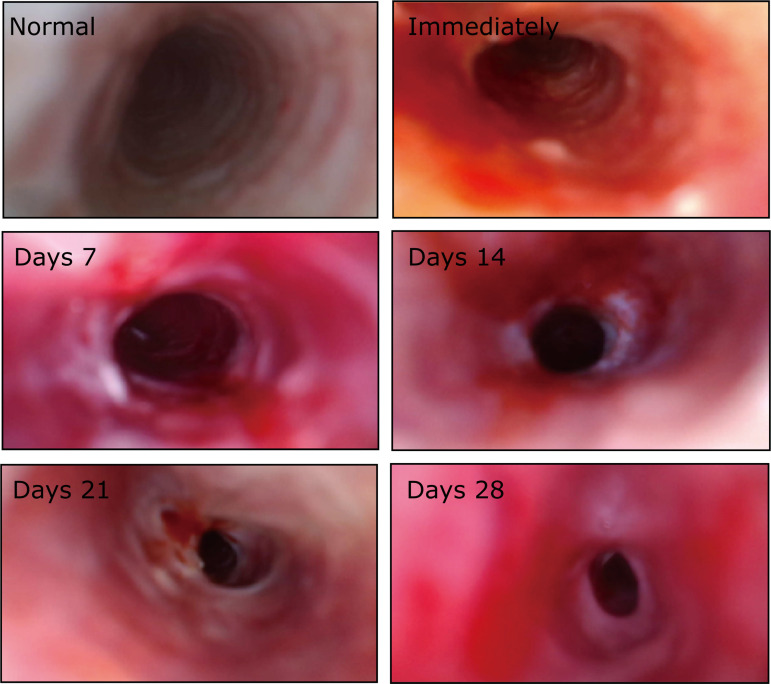
Representative images of tracheal cavities in the model group under the handy endoscope. Normal = normal tracheal lumen; Immediately = immediately after intervention; Days x = x days after intervention.

As expected, satisfying images with adequate resolution for endotracheal observation can be obtained by the household endoscope (Fig. 3, Normal), and no cyanosis, endotracheal hemorrhage and other complications were observed in the experimental rabbits during the endoscopic intervention. In the model group, endoscopy showed that the inner layer of the trachea was exfoliated immediately after scraping and the cartilage was vaguely visible (Fig. 3, Immediately). Seven days after scraping, granulation hyperplasia with high edema and congestion, accompanied by white purulent secretion, could be seen covering the wound (Fig. 3, Days 7). Granulation hyperplasia reached a peak 14 days after the intervention, and severe stenosis of the lumen appeared (Fig. 3, Days 14). Then, at 21 days postoperatively, when granulation tissue edema gradually subsided, the tissue became pale and smooth and tend to turn into a scar morphology with a resultant slight reduction of stenosis (Fig. 3, Days 21). At 28 days postoperatively, the hyperplastic tissue presented a contracture gray-white mature scar-like appearance, and lumen stenosis reached its peak during the whole observation period (Fig. 3, Days 28).

**Figure 4:**
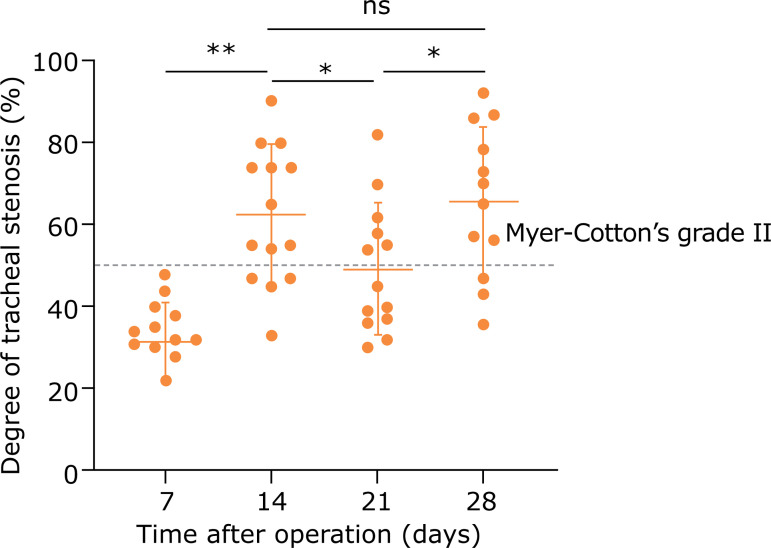
Quantification of tracheal stenosis degrees in the model group at different time points after intervention. Each dot represents data from one animal. Data are presented as mean ± s.d. (ns, nonsignificant; *p < 0.05; **p < 0.01, Student t-test).

More specifically, as shown in Fig. 4, the degree of tracheal stenosis gradually increased during the first 2 weeks, and at 14 days postoperatively, it showed a range of 33.2 – 90.4%, with a mean of 62.4%, and degree above Myer–Cotton’s grade II accounting for 71.4% (10/14). Afterward, the stenosis degree decreased slightly for a time and then increased to the peak over the study period at 28 days postoperatively, showing a range of 36.0 – 92.1% and a mean of 65.9%, and degree above Myer–Cotton’s grade II accounting for 75.0% (9/12). No abnormal hyperplasia and tracheal stenosis were found in the mock group (data not shown).

### Gross and histological findings

**Figure 5:**
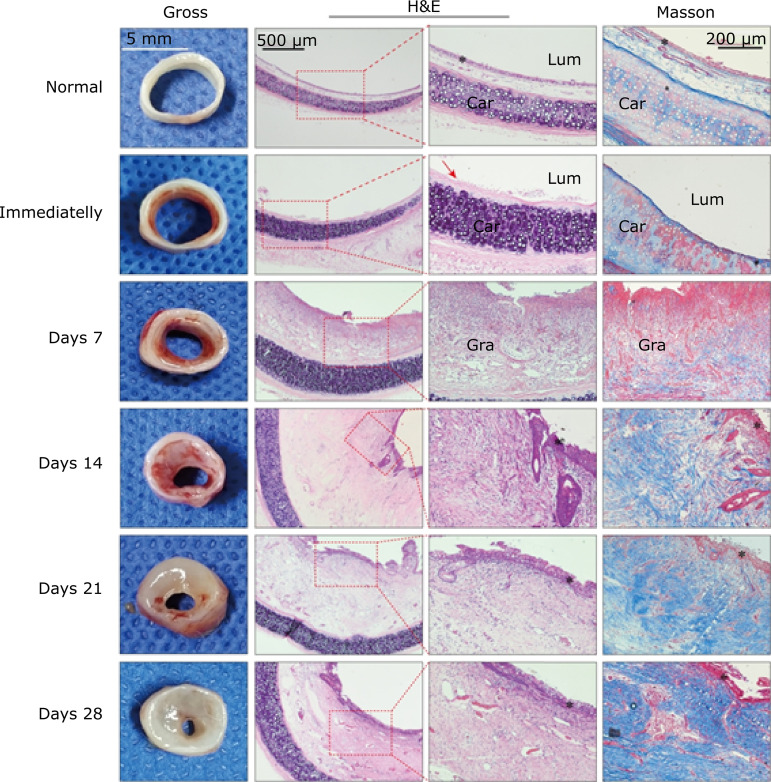
Gross and histological findings of the model group at different time points after intervention (cross-section). Gross, gross examination; H&E, hematoxylin and eosin staining; Masson, Masson’s trichrome staining (collagen fibers were stained blue, and the background was stained red). Lum, tracheal lumen; Car, cartilage; Gra, granulation tissue; red arrow, perichondrium; asterisk, epithelium.

The transverse diameter and sagittal diameter of the normal trachea of New Zealand rabbits in this study was approximately 8 and 6 mm, respectively. The tracheal wall (Fig. 5, Normal) comprised a mucosal layer including ciliated cells, goblet cells, basal cells, and brush cells; a submucosal layer containing mucus glands and serous glands; and an adventitia referring to the cartilage surrounded by the perichondrium. Obviously, these histological manifestations are analogous to those of humans. Pathological examination immediately after scraping (Fig. 5, Immediately) showed that the mucosa and submucosa above were scraped off and the perichondrium was exposed, but the adventitia was not damaged. At 7 days postoperatively (Fig. 5, Days 7), fresh and delicate granulation hyperplasia consisting of a large number of new thin-walled capillaries, proliferating fibroblasts and inflammatory cells mainly composed of neutrophils and macrophages appeared in the lumen. The granulation hyperplasia gradually expanded outward and covered the wound surface, with its surface covered with white purulent secretions rather than mucosal epithelium. Additionally, Masson staining showed that collagen began to deposit, but the arrangement was disordered. At 14 days postoperatively (Fig. 5, Days 14), granulation tissue reached its maximum volume, resulting in severe lumen stenosis. Proliferative basal cells and metaplasia squamous epithelia migrated gradually and covered the surface of the hyperplastic tissue. Although there are still many dilated and congested capillaries in the superficial layer of granulation tissue, the deep capillaries have begun to degenerate. Meanwhile, fibroblasts showed explosive growth and collagen fibers were deposited and interlaced in large quantities. At 21 days postoperatively (Fig. 5, Days 21), as the edema of the granulation tissue subsides, it becomes pale and smooth. Microscopically, the granulation tissue is covered by a layer of irregular stratified cuboidal epithelium. The interstitial edema of granulation tissue was decreased, inflammatory cell infiltration was significantly markedly reduced, and part of the capillaries were occluded and reconstructed into arterioles and venules. Meanwhile, the number of fibroblasts decreased, their cell bodies shrank with their nuclei became smaller and hyperchromatic, and transformed into fibrocytes. However, collagen fibers continued to increase and arranged regularly. In this phase, granulation tissue gradually turned into fibrous connective tissue composed of a large number of collagen fibers. At 28 days postoperatively (Fig. 5, Days 28), an annular-constricted gray-white scar was noted in the tracheal lumen, which was rigid and inelastic. Microscopically, the surface of the mucosa is largely covered by ciliated columnar cells, interspersed with goblet cells, but this epithelium layer is thicker and partially folded compared to the normal trachea mucosa. In the submucosa, there were few fibroblasts and capillaries, and only a few scattered inflammatory cells were found. The collagen fiber bundles were dense and arranged parallel to the tracheal ring. No inflammatory cell infiltration and destruction were observed in the perichondrium and cartilage, and only mild subperichondrium chondrocyte proliferation was observed.

## Discussion

In the selection of animals for the establishment of benign airway stenosis models, porcine^10^, canines^11^, rabbits^6^, rats^12^ and so on have been reported. However, the New Zealand rabbit has been the “workhorse” in the production of benign airway stenosis models due to some of its advantages: (1) Compared with large animals, such as canines, pigs, and sheep, rabbits are mild creatures that are easy to raise, harmless, and inexpensive, and can easily meet the demand of sample size; (2) Rabbit experimental reagents and equipment are advanced and complete, and related experimental procedures are pervasive; (3) Previous study has shown that the histological features of rabbit trachea and the characteristics of the healing process of the trachea after injury are similar to those in humans^13^; (4) In contrast to rabbits, which have a moderate tracheal diameter that can accommodate existing fine bronchoscope, smaller animals, such as rats, have extremely small tracheal lumens to operate and poorly tolerate and unable to perform endoscopic observation *in vivo*, and limited studies can be conducted after modeling.

Currently, one or more combined methods by open or endoscopic approach to establish rabbit model of tracheal stenosis have been reported^5^,^14^, such as nylon brush or spur scraping^4^,^6^,^15^, chemical injury, electrocautery^5^, laser cautery^16^, stenting^7^, and segmented tracheal catheterization^8^. Among them, nylon brush scraping, as a classical modeling technique, has been widely adopted. It is usually achieved with the aid of a medical endoscope or via a tracheotomy^4^. However, this may lead to an occupation of delicate medical endoscope, which is relatively expensive and not readily accessible, or involve annoying tracheotomy complications, including the introduction of interference factors, severe trauma, increased risk of infection, longer recovery time, increased postoperative care, etc. To address this issue, a modified modeling method that required only two steps, including transoral intubation and scraping the tracheal mucosa by introducing a nylon brush through the catheter is developed. This method is rapid, minimally invasive and cost-effectively, with fewer complications and satisfying survival rate, acceptable model stability and high stenosis degree. Above all, it reduced requirements of both elaborate techniques and expensive equipment, which is a boon for budding researchers.

To achieve it, several hurdles have been crossed. The first hurdle is achieving orotracheal intubation in rabbits. The rabbit glottis is difficult to directly visualize due to their relatively large tongue and small oropharyngeal cavity, and the vision on the glottis can be further obstructed when the front end of a tracheal catheter enters the mouth. Therefore, in most cases, blind orotracheal intubation has to be performed^17^. Gratifyingly, this issue was overcome by achieving a straight view of the glottis under a neonatal laryngoscope and then passing a fine guide wire through the glottis and guiding a tracheal catheter into the trachea. This technique has a high success rate, considerably shortens the intubation time, and reduces intubation-derived complications. It is worth mentioning that the role of intubation is to quickly and accurately guide the nylon brush to the trachea and avoid additional injury on the entry route of the brush. It does not cause any additional damage that would have led to stenosis, which was ruled out in the mock group during follow-up.

Another hurdle is to prevent hypoxia and bleeding-induced asphyxia during intervention. In fact, no hypoxic symptoms were observed in the model group during the intervention. One explanation is that the sparse bristles allow ventilation and the scraping time is too short to affect the rabbits’ oxygen supply. Although tracheal hemorrhage in animals after tracheotomy is relatively easy to address, it was not a thorny issue in previous studies of nontracheotomy modeling methods^4^,^5^,^15^. In this study, again, that was not a trouble, as some optimizations were done to prevent airway bleeding-induced asphyxia, including postural drainage, negative pressure suction, and light anesthesia that maintained a moderate cough reflex during intervention and allowed rapid postoperative resuscitation to cough up possible blood clots. In fact, there was only minimal bleeding followed by rapid spontaneous hemostasis, and there were no deaths due to intraoperative or postoperative airway bleeding in the model rabbits.

The biggest hurdle is to achieve homogeneous injury to the tracheal wall at each modeling intervention to enhance the stability and reproducibility of the animal model. Considering the size of the New Zealand rabbit’s trachea and the injury depth desired, a suitable nylon brush was used and an optimized scraping procedure was adopted, so that the scraping just scraped off the relatively soft tissue above the rigid perichondrium and tracheal membranous ligament to control the depth of the injury, which was also confirmed by pathological examination immediately after scraping. However, complicated influencing factors together with individual differences make it difficult to obtain models of the same stenosis even if their tracheas suffer the same injury.

Previous study has also shown that there is no method for inducing tracheal stenosis model that can reliably predict the severity of stenosis or produce uniform stenosis^4^. Moreover, the grade of tracheal stenosis is more closely related to prognosis and treatments than its absolute degree. Therefore, for comparative studies, it may be more practical to group the models by grade (e.g., using the Myer–Cotton’s grading system) rather than by absolute degree to ensure that each group is comparable^9^,^15^. According to relevant reports, which showed stenosis ranged from 10 to 90% with a mean of 43%, the range of models in this study is relatively concentrated, and the mean stenosis degree is also at a relatively high level^4^,^15^.

As for the depth of injury, a concept researchers agreed upon is that simple tracheal mucosal injury results in complete mucosal regeneration without causing luminal stenosis^14^. Some researchers even affirm that the key to cause tracheal stenosis is the injury of the perichondrium nourishing the cartilage, which can fairly cause degenerative necrosis, deformation, and collapse of the cartilage bracket, leading to the development of stenosis, especially severe stenosis^5^,^14^. However, this study suggests that injury to the perichondrium is unnecessary for stenosis or even severe stenosis, and adequate damage to the submucosa also produce markedly thickened fibrotic repair tissue, leading to severe tracheal stenosis. Furthermore, for animal models, a high degree of stenosis often implies a high mortality, and the early onset of fatal stenosis leaves the model without a complete period of observation. models in this study showed adequate stenosis degree, but a relatively low mortality that most models can survive until stable stenosis is formed, thus, moderate injury, such as nylon brush scraping to the trachea, may sometimes be more expedient. In fact, whether damage of the perichondrium and cartilage is involved in modeling depends more on the clinicopathological features to be reproduced in the model. After all, clinically, not all airway stenosis involved an abnormal cartilage. Besides, it is worth mentioning that in this modeling method, the injury area can be adjusted by changing the length of the brush; Likewise, the depth of injury can also be adjusted theoretically by enhancing the hardness of the bristles, increasing the outside diameter of the brush, or increasing scraping times.

The clinicopathological process of common tracheal stenosis secondary to mechanical injury caused by tracheal intubation, tracheal surgery, or bronchoscope interventional therapy is similar, including a series of complex, orderly, and overlapping stages: inflammatory reaction stage, granulation tissue hyperplasia stage, and scar formation stage^18^. This study depicts, in greater detail than previously reported, the pathological course and degree changes during the formation of tracheal stenosis caused by mechanical injury to rabbits. The results again confirmed that the healing characteristics of the rabbit trachea after injury were extremely similar to that of humans, and it was an ideal animal model for the study of benign tracheal stenosis. Additionally, it should be noted that the stenosis at different pathological stages has varying pathological characteristics, involving different cell compositions, cytokines, and other driving factors, requiring different treatment regimens. Moreover, in evaluating the effectiveness of therapeutic interventions, care should be taken to distinguish whether the improvement in pathological findings is due to interventions or simply a natural pathological change. Herein, this modeling method can meet the needs for models at different pathological stages, and the detailed description of the pathological process also provides a reference for experimental design based on this model.

Undoubtedly, it is very important to monitor and evaluate the stenosis in the modeling process or related studies of tracheal stenosis models, based on which subsequent grouping could be performed, determine the timing of intervention or compare the efficacy of different treatments. Nevertheless, follow-up examinations of the models by radiography, computed tomography, or medical bronchoscopy are inconvenient, not readily accessible, and costly in many research institutions, especially when frequent examinations are required. To confront this dilemma, an idea of utilizing a handy and low-cost endoscope came up. A household endoscope, with an outer diameter of 3.9 mm and cost < ¥ 1,000 (approximately US$ 150), was found just right for safely inserting the rabbit’s trachea and could obtain images with adequate discriminating information for the evaluation of endotracheal lesions. It is portable and, most importantly, low-cost, providing an easily accessible and useful tool for facilitating broader research into benign tracheal stenosis, especially in numerous developing countries that lack facilities and funding. Furthermore, given its relatively simple performance, some optimizations were made to enhance its usability, such as using a preintubated catheter to guide the endoscope smoothly into the trachea, manually adjusting the curvature of the rabbit cervical spine so that the lens is always in the center of the tracheal, and preheating the lens with warm water to prevent airway steam from fogging the lens. Of course, this endoscope would also work in animal models with larger tracheal diameters, which would extend its application.

Unfortunately, with regard to this modeling method, there are still some drawbacks: (1) This model can only reproduce the pathogenesis of human benign tracheal stenosis caused by mechanical injury, excluding congenital defect, infectious disease, idiopathic etiology, and benign airway tumors, and its rationality and completeness are imperfect due to species difference between models and humans so that research evidence can only be adopted to a limited extent; (2) As the degree of tracheal stenosis in the models are still remarkably uncontrollable and unpredictable, how to further improve its performance and control the potential influencing factors to create a more stable and uniform model is still a major challenge; (3) In addition to pathological course, it is necessary to verify the expression profile of cytokines related to inflammation and fibrosis during the formation of the stenosis to further confirm the effective interpretation of the animal model presented here; (4) This study did not explore the effect of a deeper injury or a longer segmental injury to the tracheal wall on the repair outcome. Does the injury to the perichondrium or cartilage activate a specific inflammatory response and induce fibroblasts with more fibrotic characteristics, thus aggravating the stenosis other than the deformation and collapse of the cartilage bracket? Does scraping a longer segment of the trachea correspondingly lead to longer repair periods, resulting in a more persistent inflammatory response in the wound center, which in turn exacerbates the eventual stenosis? Further studies are needed to obtain a definitive answer.

## Conclusions

The modified rabbit model of tracheal stenosis is simple, minimally invasive, and reliable. The low-cost household endoscope is competent for follow-up evaluation of the model. These two simple and accessible tools can promote extensive studies of benign tracheal stenosis.
